# Developmental Expression of Mutant PFN1 in Motor Neurons Impacts Neuronal Growth and Motor Performance of Young and Adult Mice

**DOI:** 10.3389/fnmol.2019.00231

**Published:** 2019-09-27

**Authors:** Merryn Brettle, Holly Stefen, Aleksandra Djordjevic, Sandra Y. Y. Fok, Josephine W. Chan, Annika van Hummel, Julia van der Hoven, Magdalena Przybyla, Alexander Volkerling, Yazi D. Ke, Fabien Delerue, Lars M. Ittner, Thomas Fath

**Affiliations:** ^1^School of Medical Sciences, Faculty of Medicine, UNSW Sydney, Randwick, NSW, Australia; ^2^Biomedical Imaging Facility, Mark Wainwright Analytical Centre, UNSW Sydney, Randwick, NSW, Australia; ^3^Dementia Research Centre and Department of Biomedical Sciences, Faculty of Medicine and Health Sciences, Macquarie University, Sydney, NSW, Australia

**Keywords:** profilin 1, amyotrophic lateral sclerosis, actin, neurodegeneration, motor neurons

## Abstract

Amyotrophic lateral sclerosis (ALS) is a devastating neurodegenerative disease with limited treatment and no cure. Mutations in *profilin 1* were identified as a cause of familial ALS (fALS) in 2012. We investigated the functional impact of mutant profilin 1 expression in spinal cords during mouse development. We developed a novel mouse model with the expression of profilin 1 C71G under the control of the *Hb9* promoter, targeting expression to α-motor neurons in the spinal cord during development. Embryos of transgenic mice showed evidence of a significant reduction of brachial nerve diameter and a loss of Mendelian inheritance. Despite the lack of transgene expression, adult mice presented with significant motor deficits. Transgenic mice had a significant reduction in the number of motor neurons in the spinal cord. Further analysis of these motor neurons in aged transgenic mice revealed reduced levels of TDP-43 and ChAT expression. Although profilin 1 C71G was only expressed during development, adult mice presented with some ALS-associated pathology and motor symptoms. This study highlights the effect of profilin 1 during neurodevelopment and the impact that this may have in later ALS.

## Introduction

Amyotrophic lateral sclerosis (ALS) is the most common form of motor neuron disease. The disease is characterized by progressive neurodegeneration, causing paralysis and death (Gordon, 2013). Approximately 10% of cases are familial with an average age of onset 10 years earlier than sporadic ALS (sALS; Chiò et al., 2009). Despite this difference in age of onset, familial ALS (fALS) and sALS present with clinically comparable features (Neumann et al., 2006; Swarup et al., 2011; Renton et al., 2014). Mutations in the profilin 1-encoding *PFN1* gene were identified as a rare cause of ALS in 2012 (Wu et al., 2012). The study by Wu et al. (2012) identified four mutations in the *PFN1* gene; C71G, M114T, E117G and G118V. Subsequent studies have identified additional mutations in both sALS and fALS including A20T, T109M, Q139L and R136W (Chen et al., 2013; Ingre et al., 2013; Smith et al., 2015; Yang et al., 2016). PFN1 is historically known as an actin-binding protein (Witke et al., 1998). However, more recent studies have highlighted the importance of PFN1 as a potential regulator in the dynamics of both actin and microtubules (Nejedla et al., 2016; Henty-Ridilla et al., 2017).

The complexity of ALS is highlighted by the number of genes that have been directly implicated in cases of fALS. To date, mutations in 30 genes have been identified as a cause of fALS, including mutations in the *SOD1* and *TARDBP* genes, with some mutations more common than others (Rosen et al., 1993; Sreedharan et al., 2008; Leblond et al., 2014; Renton et al., 2014; Chia et al., 2018). Although mutations in *PFN1* are the etiological cause of less than 1% of fALS, understanding the role of PFN1 in disease has the potential to inform about the pathogenesis of sALS and assist in therapy development. It is currently unknown how mutations in a variety of functionally different genes can all converge to cause ALS.

PFN1 is an actin-binding protein with ubiquitous expression (Kwiatkowski and Bruns, 1988). PFN1 mediates the exchange between ADP-actin and ATP-actin, therefore enhancing actin filament extension (Mockrin and Korn, 1980; Wen et al., 2008). The regulation of PFN1 expression is important, as at high cellular concentrations, PFN1 can inhibit actin filament extension (Courtemanche and Pollard, 2013). PFN1 also controls filamentous actin by regulating the localization of actin monomers (Vitriol and Zheng, 2012; Lee et al., 2013). In addition to its traditional role in actin dynamics, PFN1 has been shown to impact on microtubule dynamics, hence forming a regulatory link between the complex interplay of the two major cytoskeletal systems (Nejedla et al., 2016; Henty-Ridilla et al., 2017; Dogterom and Koenderink, 2019). To date, two mouse models, expressing ALS-associated PFN1 mutations have been reported (Yang et al., 2016; Fil et al., 2017). Both studies detail mouse models that exhibit classical ALS phenotypes of progressive motor deficits, resulting in paralysis and a significant impact on survival. Yang et al. (2016) presented a triple transgenic *Thy1.2* PFN1^C71G/C71G^/Prp PFN1^C71G^ mouse with an aggressive ALS phenotype. They utilized the *Thy1.2* promoter and prion promoter to drive pan-neuronal and ubiquitous expression of PFN1^C71G^ (Yang et al., 2016). The second mouse model used a prion promoter for the expression of PFN1^G118V^ (Fil et al., 2017). Both studies produced control mice overexpressing PFN1^WT^ and observed no significant changes between PFN1^WT^ and control mice (Yang et al., 2016; Fil et al., 2017). To this end, however, it remains unclear what effects of mutant PFN1 on α-motor neuron dysfunction can be attributed to its expression during development vs. those effects resulting from its disrupted function in mature neurons later in life.

Changes to α-motor neuron morphology and electrophysiology occur much earlier in ALS pathology than previously thought, as does α-motor neuron death (Avossa et al., 2006; Martin et al., 2013; Vinsant et al., 2013a,b). This indicates that morphological alterations during neurodevelopment may impact the electrophysiological properties of developing α-motor neurons and potentially prime them for an early neuronal death. Here, we have utilized an α-motor neuron-specific *Hb9* promoter to drive PFN1^C71G^ expression only during development (Arber et al., 1999). Driving the transgene expression with the *Hb9* promoter controls not only the spatial but also the temporal localization of the transgene. The temporally restricted expression of the mutant PFN1 in our mouse model allows us to interrogate whether there is a contribution of the early expression of mutant PFN1 to ALS disease pathology. We characterized PFN1 expression in the novel mouse model and studied the impact on neurodevelopment. We hypothesized that changes during development could lead to morphological changes in the spinal cord of adult mice, resulting in functional changes. To investigate this, we performed motor testing of both young and aged transgenic mice. Finally, we studied the molecular consequence of PFN1^C71G^ expression by looking for evidence of ALS pathology in the spinal cords of aged mice.

## Materials and Methods

### Animals

All animal procedures were conducted in accordance with the Animal Care and Ethics Committee of UNSW Sydney. Mice were housed on a 12 h light/dark cycle. V5-hPFN1^C71G^ was cloned into the *Pme*I and *Spe*I sites of the p*Hb9*-MCS-IRES-EGFP plasmid (*mHb9* promoter) [TJ#103], which was a kind gift from Thomas Jessell (Addgene plasmid #16283; Wilson et al., 2005). A fragment, containing the *mHb9* promoter, the sequence for V5-hPFN1^C71G^, and an IRES site followed by the sequence for EGFP was used for the generation of the *Hb9* V5-PFN1^C71G^ transgenic mouse line. The transgenic mice were generated on a C57Bl/6 background by pronuclear injection (Ittner and Gotz, 2007; Delerue and Ittner, 2017).

For the analysis of motor performance, C57Bl/6 Wild type (Wt) littermates were used as controls. Rotarod performance was analyzed as previously described (Ke et al., 2015). An acceleration mode was used with the speed increasing from 7 to 60 rpm over a 120 s period on a 5-wheel Rotarod treadmill (Ugo Basile). Latency to fall was measured and recorded. Each mouse was given four attempts and the best attempt was recorded. Forelimb grip strength was measured with a grip strength meter (Ametek). Each mouse was given five attempts and the highest peak force (N) exerted was recorded (Ittner et al., 2016). The hanging wire test was used as previously described (van Hummel et al., 2018). For this, mice completed two trials and the best performance was recorded.

Any deaths of *Hb9* V5-PFN1^C71G^ mice were recorded with the date and cause of death. If the condition of mice deteriorated, the mice were euthanized, in accordance with ethics approval from the Animal Care and Ethics Committee at UNSW Sydney. Mice that were euthanized, or that were found dead in their cages, were included in the analysis of survival data.

### Immunohistochemistry

For the collection of embryos, C57Bl/6 females were bred with *Hb9* V5-PFN1^C71G^ ^Wt/Tg^ males, using a timed mating protocol, where the time of plug check was considered to be E0.5. Pregnant females were euthanized by cervical dislocation at the appropriate timepoint. Embryos were harvested and immersion fixed in ice-cold 4% paraformaldehyde (PFA) for 90–120 min. Adult mice were anesthetized and transcardially perfused with ice-cold 1× phosphate-buffered saline (PBS). Brains were collected and fixed overnight in 4% PFA at 4°C.

The tissues were processed with an ethanol and xylene gradient in an Excelsior tissue processor (Thermo). Whole embryos were embedded in paraffin blocks. Spinal cords were cut into 2 mm segments and embedded into paraffin blocks with cervical to sacral sections arranged in a grid-like pattern. Samples were sectioned with a 5 μm thickness on a microtome (Leica). Sections were rehydrated and stained as previously described (Ittner et al., 2016). Antibodies used in this study were: anti-ChAT (Millipore), anti-profilin 1 (Abcam), anti-tau1 (Millipore), anti-TDP-43 (Proteintech) and anti-V5 (Invitrogen, Carlsbad, CA, USA).

### Wholemount Staining and Lightsheet Microscopy

Embryos were collected as above and stained as previously described (Wurdak et al., 2005). Embryos were mounted in low melting temperature agarose (Sigma-Aldrich, St. Louis, MO, USA). Prior to imaging, embryos were cleared in CUBIC reagent 2 for 24 h with gentle agitation. The embryos were imaged on a Zeiss Lightsheet Z.1 microscope with a 5× clearing objective. The imaging chamber was filled with CUBIC reagent 2 to match the refractive index of the cleared specimen.

### Image Collection and Analysis

Fluorescence imaging of sections was completed on either a BX51 (Olympus) or Axioskop 40 (Zeiss) epifluorescence microscope. Brightfield images were captured on a BX51 (Olympus) microscope with a white balance filter. All image analysis was performed with Fiji [(Fiji is just) ImageJ 2.0.0-rc-43/1.52d, National Institutes of Health; Schindelin et al., 2012]. Adobe Photoshop and Illustrator were used to prepare images and figures.

### Analysis and Statistics

For behavioral tests, data were collected by staff, blinded to genotypes of experimental groups. Data were organized on Excel (Microsoft) and analyzed in GraphPad Prism software (version 6.0, Graphpad software). Normality of data was assessed using D’Agostino and Pearson omnibus normality test. Data with two groups that had Gaussian distribution was analyzed by student’s *t*-tests and data with non-Gaussian distribution were analyzed by a Mann–Whitney test. Data with more than two groups and across multiple time points were analyzed with two-way analysis of variance (ANOVA). Mendelian inheritance was analyzed with Chi-squared analysis and the appropriate degree/s of freedom. Survival data were analyzed with a log-rank (Mantel-cox) test.

## Results

### *Hb9* V5-PFN1^C71G^ Transgenic Mice Express PFN1^C71G^ in the Spinal Cord Embryonic During Development

**Figure 1:**
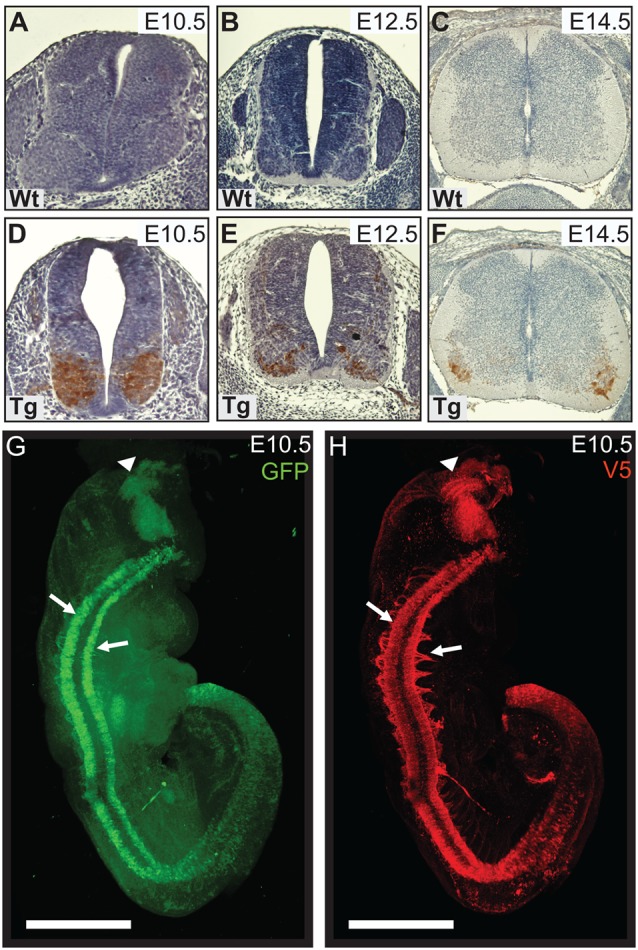
Wild type control (Wt) and transgenic (Tg) tissues were stained for V5-DAB. Wt embryos have no expression of V5-PFN1^C71G^ **(A–C)**. V5-PFN1^C71G^ positive staining can be seen in the basal plate of the neural tube of Tg E10.5–14.5 embryos **(D–F)**. Whole E10.5 embryos were stained for GFP and V5-PFN1^C71G^ and imaged on a Zeiss Lightsheet Z.1 microscope. The rendered images show GFP (green) and V5 (red) expression in a Tg embryo **(G,H)**. V5-PFN1^C71G^ expression is present throughout the length of the neural tube in the lateral basal plates (arrows), in the axonal bundles extending from the neural tube, and in the mid-hindbrain junction of the developing brain (arrowhead). Scale bars = 500 μm.

We generated a transgenic mouse line with a murine *Hb9* promoter to test whether early expression of mutant PFN1 in motor neurons has a detrimental effect on motor neuron development and function. *Hb9* is a homeobox gene that determines the fate of α-motor neurons, with expression switching on between embryonic day 9.5 (E9.5) and E10.5. Accordingly, the *Hb9* promoter restricts expression to α-motor neurons during specific developmental stages (Arber et al., 1999). We characterized the temporal expression of V5-PFN1^C71G^ in transgenic embryos. No staining was observed in Wt controls (Figures 1A–C) or in E9.5 transgenic embryos (data not shown). V5-positive staining is apparent in the basal plates of the spinal cords of E10.5, E12.5 and E14.5 transgenic embryos (Figures 1D–F). This is consistent with the published literature on the promoter activity (Arber et al., 1999). In P0 transgenic pups, expression of PFN1^C71G^ is lower than the expression in sections from transgenic embryos (Supplementary Figure S1). There is no expression of PFN1^C71G^ in the brain or spinal cords of adult *Hb9* V5-PFN1^C71G^ transgenic mice from 1 month to 18-month-old (Supplementary Figure S1).

We utilized Lightsheet imaging to characterize the spatial expression of EGFP (green) and V5 (red) in E10.5 transgenic embryos (Figures 1G,H). EGFP and V5-PFN1^C71G^ expression was observed throughout the length of the neural tube (arrows) and in the mid-hindbrain junction of the developing brain (arrowhead; Figures 1G,H). Expression of V5-PFN1^C71G^ appeared to extend further into the developing axons than EGFP (Figures 1G,H). This localization is consistent with the role of PFN1 in neurite extension.

**Figure 2:**
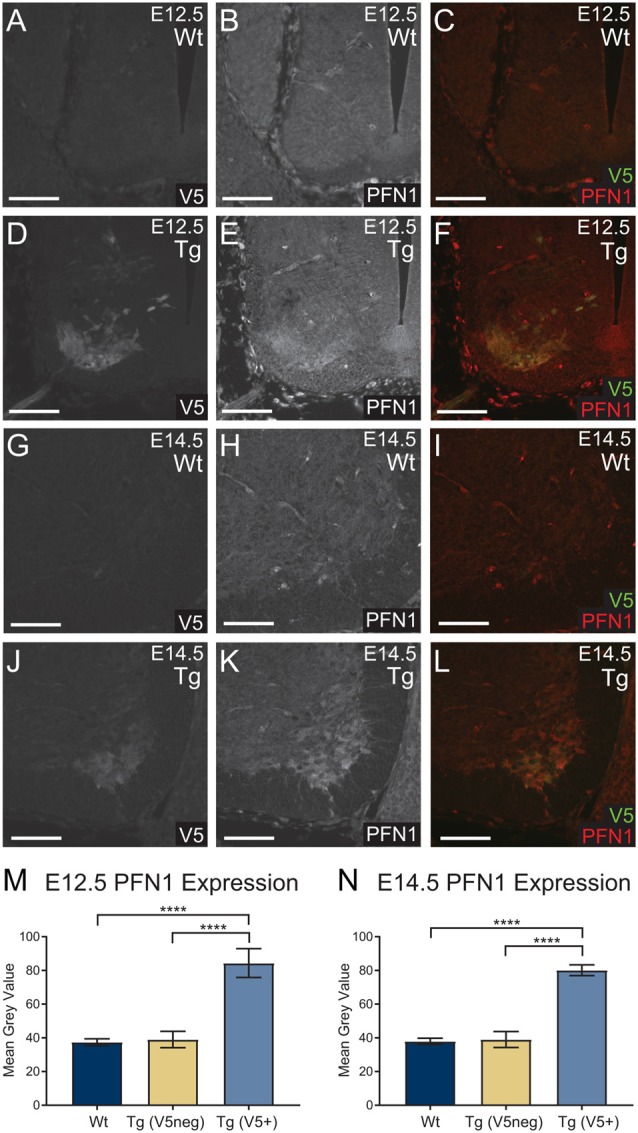
Wild type control (Wt) and transgenic (Tg) sections were stained for V5 (green) and PFN1 (red). No V5 expression or PFN1 overexpression was detected in Wt embryos **(A–C,G–I)**. Sections from Tg E12.5 and E14.5 embryos had V5 expression and PFN1 overexpression in the anterior portion of the neural tube **(D–F,J–L)**. Analysis of Tg embryos was split into V5 negative and V5 positive areas. Tg E12.5 and E14.5 embryos had a significant increase of PFN1 in V5 positive areas when compared to V5 negative areas in Tg and Wt embryos **(M,N)**. Analyzed with one-way analysis of variance (ANOVA; **M,N**); ****p ≤ 0.0001; graphed with SEM. Scale bars = 50 μm.

To assess the level of PFN1^C71G^ expression, we analyzed PFN1 intensity in V5-positive tissue. Sections from E12.5 and E14.5 embryos were double immunolabeled for V5 (green) and PFN1 (red; Figures 2A–L). V5-positive regions of transgenic embryos showed a marked increase in PFN1 expression (Figures 2A–L). There was a significant increase in PFN1 intensity in V5-positive areas of neural tubes from transgenic mice when compared to PFN1 intensity in V5-negative areas of transgenic and Wt neural tubes (Figures 2M,N). The overexpression of PFN1^C71G^ resulted in a 2.11–2.25-fold increase in the total levels of PFN1 in the developing motor neurons of developing transgenic embryos.

**Figure 3:**
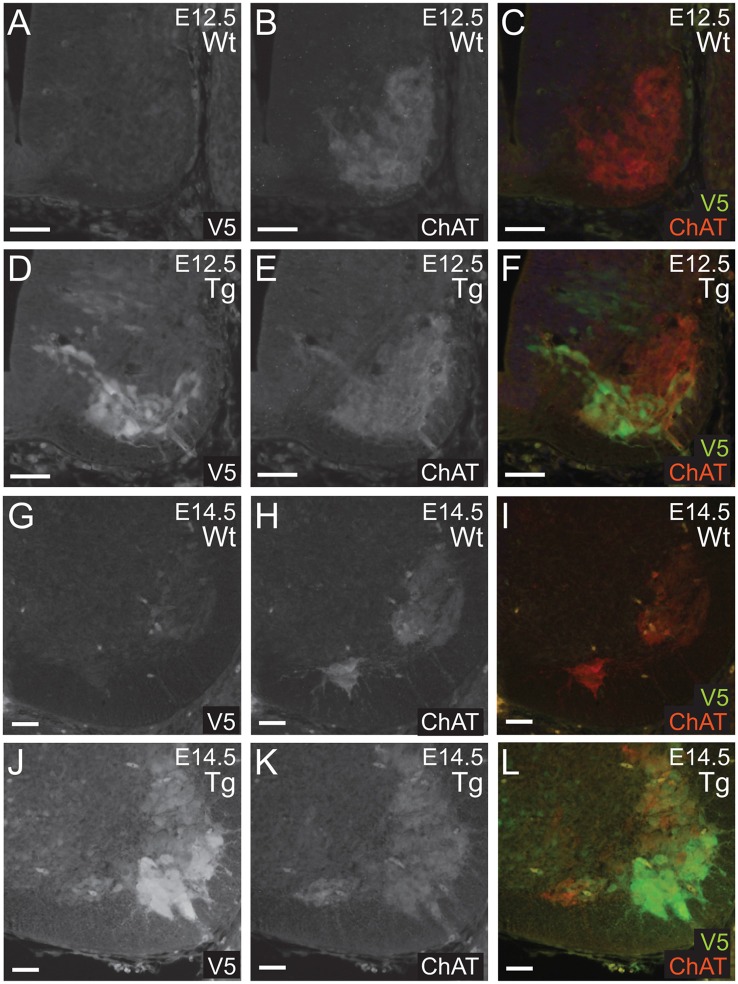
Wild type control (Wt) and transgenic (Tg) sections were stained for V5 (green) and ChAT (red). ChAT expression was detected in Wt embryos **(A–C,G–I)**. Sections from E12.5 and E14.5 Tg embryos had V5 expression in ChAT positive areas of the anterior portion of the neural tube **(D–F,J–L)**. Scale bars = 50 μm.

Next, we confirmed that PFN1^C71G^ was being expressed in developing α-motor neurons. Sections from E12.5 and E14.5 embryos were double immunolabeled for V5 (green) and ChAT (red; Figures 3A–L). Staining showed that V5-positive neurons were also positive for ChAT in sections from transgenic embryos (Figures 3A–L). This confirmed that the expression of the transgene was in the correct neuronal subtype, developing motor neurons.

### Developmental Expression of PFN1^C71G^ Reduces Brachial Nerve Diameter

**Figure 4:**
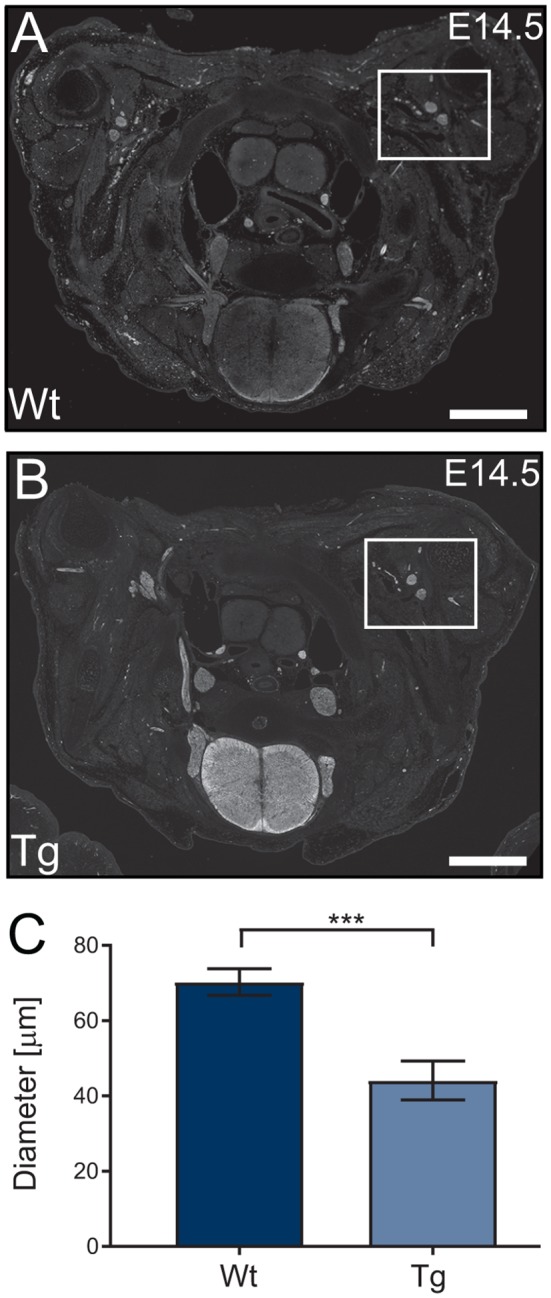
Wild type control (Wt) and transgenic (Tg) sections from Wt and Tg embryos were stained with Tau1 **(A,B)** and the diameter of the brachial nerve bundles were analyzed. Analysis revealed that Tg animals had a reduction in the diameter of brachial nerves (**C**; *p* = 0.0003). *N* = 4 animals for each genotype. Analyzed with unpaired student *t*-test; ***p ≤ 0.001; graphed with standard error of the mean (SEM). Scale bars = 500 μm.

Following the characterization of the transgene expression, we assessed the impact of PFN1^C71G^ expression on axonal outgrowth by analyzing brachial nerve diameter. Sections from E14.5 transgenic embryos were stained, using Tau1 antibody (axonal marker) to visualize developing axonal bundles. The shortest diameter was measured and analyzed. Transgenic animals had a significant reduction in brachial nerve diameter when compared to Wt controls (Figures 4A–C). Expression of PFN1^C71G^ in the developing α-motor neurons significantly impacted the formation of brachial nerves.

### Developmental Expression of PFN1^C71G^ Impacts the Motor Performance, Transgene Penetrance and Survival of *Hb9* V5-PFN1^C71G^ Transgenic Mice

**Figure 5:**
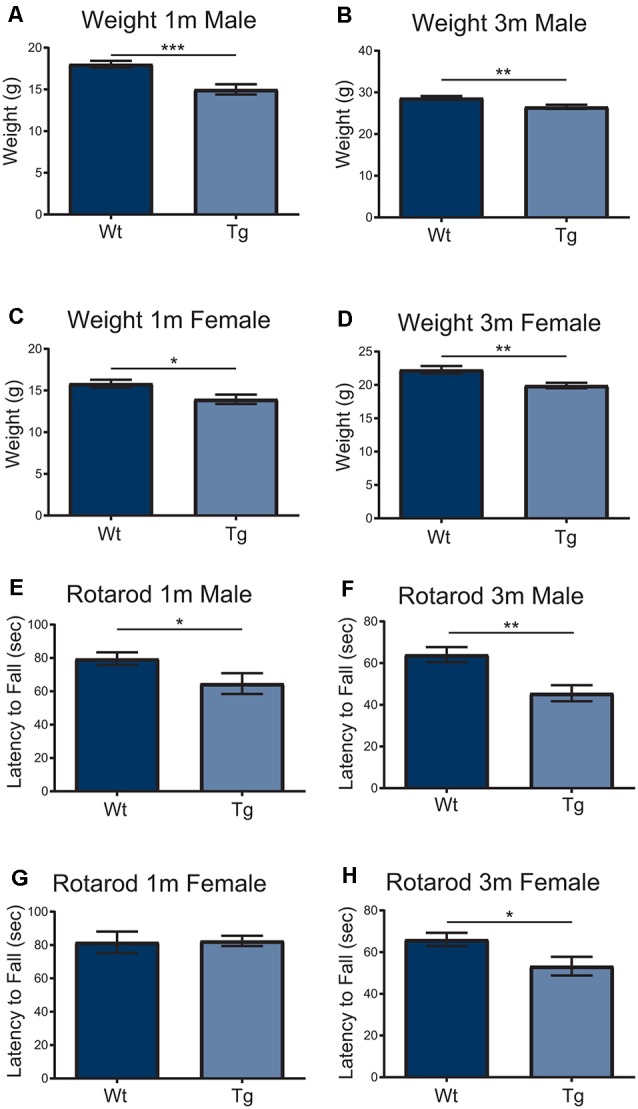
Both male and female transgenic (Tg) mice aged 1–3-months weighed significantly less than Wild type (Wt) controls **(A–D)**. Mice were challenged with an accelerating RotaRod and male Tg animals had a significant decrease in latency to fall on RotaRod at 1-month and 3-months **(E,F)**. The performance of female Tg mice was comparable to Wt at 1-month-old **(G)** but presented with a significant decrease in latency to fall at 3-months-old **(H)**. Data were analyzed with unpaired student *t*-tests; *p ≤ 0.05; **p ≤ 0.01; ***p ≤ 0.001; graphed with SEM. *N* = 6–26.

In order to understand the impact of PFN1^C71G^ during development, the *Hb9* V5-PFN1^C71G^ mouse line underwent postnatal motor testing. At 1 month and 3-month timepoints, both male and female transgenic mice weighed significantly less than the Wt controls (Figures 5A–D). When challenged on the accelerating Rotarod, 1-month and 3-month male transgenic mice fell from the treadmill significantly earlier than the Wt controls (Figures 5E,F). While 3-month-old female transgenic mice presented with a significant deficit on Rotarod when compared to Wt control, 1-month-old female transgenic mice had a fall latency that was comparable to Wt controls (Figures 5G,H). Expression of PFN1^C71G^ in developing α-motor neurons impacts the weight and motor performance of adult mice.

**Figure 6:**
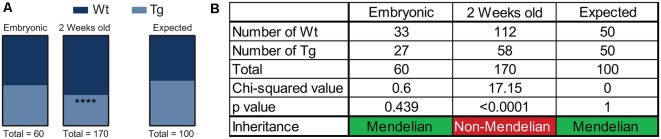
*Hb9* V5-PFN1^C71G^ mouse embryos were genotyped at the time of harvest by polymerase chain reaction (PCR) and visualization of GFP in the neural tube. Postnatal mice were genotyped by PCR at 2 weeks old. Heterozygous *Hb9* V5-PFN1^C71G^ males were bred with C57Bl/6J females. The genotype of *Hb9* embryos fits the expected Mendelian inheritance pattern **(A)**. The mice in the mouse colony were genotyped at 2 weeks of age, and no longer fit with a Mendelian inheritance pattern **(B)**. ****p < 0.0001.

Over the course of the breeding program of *Hb9* V5-PFN1^C71G^ mice, we observed that the proportion of Wt controls and transgenic mice was not consistent with Mendelian inheritance. The breeding strategy should have yielded 50% Wt controls and 50% transgenic mice. To assess this, we performed a chi-squared analysis on the proportion of mice with each genotype in both embryos and adult mice (Figures 6A,B). We found that the proportion of Wt controls and transgenic embryos fit within an expected Mendelian inheritance (Figures 6A,B). However, 65.9% of adult mice had a genotype of Wt control, with only 34.1% of adult mice with a transgenic genotype (Figures 6A,B). Therefore, expression of PFN1^C71G^ in developing α-motor neurons impacts Mendelian transgene inheritance of adult mice.

**Figure 7:**
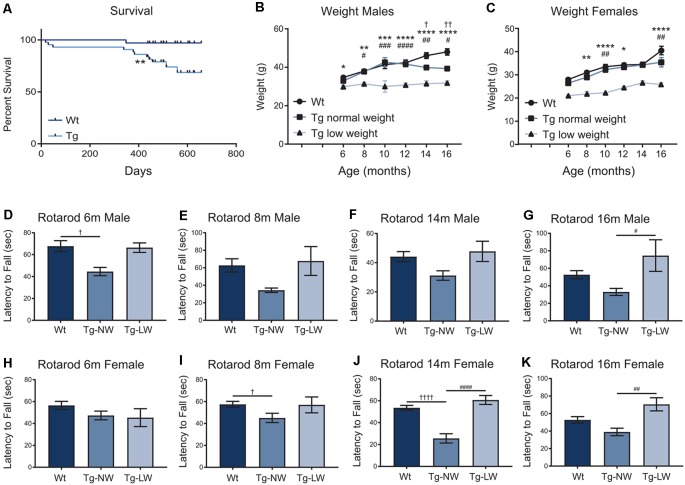
The survival of transgenic (Tg) mice was significantly impacted when compared to Wt controls **(A)**. In animals older than 6 months, transgenic (Tg) mice separated into two groups; low weight (Tg-LW) and normal weight (Tg-NW; **B,C**). *Denotes a difference between Tg-LW and Wt, ^#^denotes a difference between Tg-LW and Tg-NW, ^†^denotes difference between Tg-NW and Wt. Weight data were analyzed with two-way ANOVA; Rotarod data were analyzed with one-way ANOVA **(D–K)**; Survival data were analyzed with a Log rank test; *p ≤ 0.05; **p ≤ 0.01; ***p ≤ 0.001, ****p ≤ 0.0001; ^#^*p* ≤ 0.05; ^##^*p* ≤ 0.01; ^###^*p* ≤ 0.001, ^####^*p* ≤ 0.0001; ^†^*p* ≤ 0.05; ^†^^†^*p* ≤ 0.01; ^†^^†^^†^^†^*p* ≤ 0.0001; graphed with SEM. *N* = 3–21.

As mice were aged, additional weight and motor performance data were collected. Transgenic mice had a significantly decreased survival rate when compared with Wt controls (Figure 7A). From 6 m of age, an unusual split phenotype was revealed in both the weight and the motor performance of *Hb9* V5-PFN1^C71G^ transgenic mice. Transgenic mice older than 6 months separated into two significantly distinct weight groups; low weight and normal weight (Figures 7B,C). The low weight transgenic mice plateaued in weight from 6 months old, while the normal weight transgenic mice had weights that were comparable to Wt controls (Figures 7D–K). When examining the Rotarod results of the two different phenotypes of transgenic mice another clear trend emerged. Surprisingly, only the normal weight transgenic mice showed significant motor deficits when compared to Wt controls and their low weight transgenic littermates (Figures 7D–K). Not all age groups showed significant differences, this is due to a reduction of power in the statistical tests due to the split phenotype. The split phenotype reduced the *n* number of transgenic mice for the analysis. Another confounding factor was the impact of transgene expression on the survival of *Hb9* V5-PFN1^C71G^ transgenic mice in both the low and normal weight group. Taken together these data show that expression of PFN1^C71G^ in developing α-motor neurons impacts the survival, weight and motor performance of aged mice.

### Aged *Hb9* V5-PFN1^C71G^ Transgenic Mice Showed Evidence of Motor Neuron Loss and a Reduction of TDP-43 Expression in Motor Neurons

**Figure 8:**
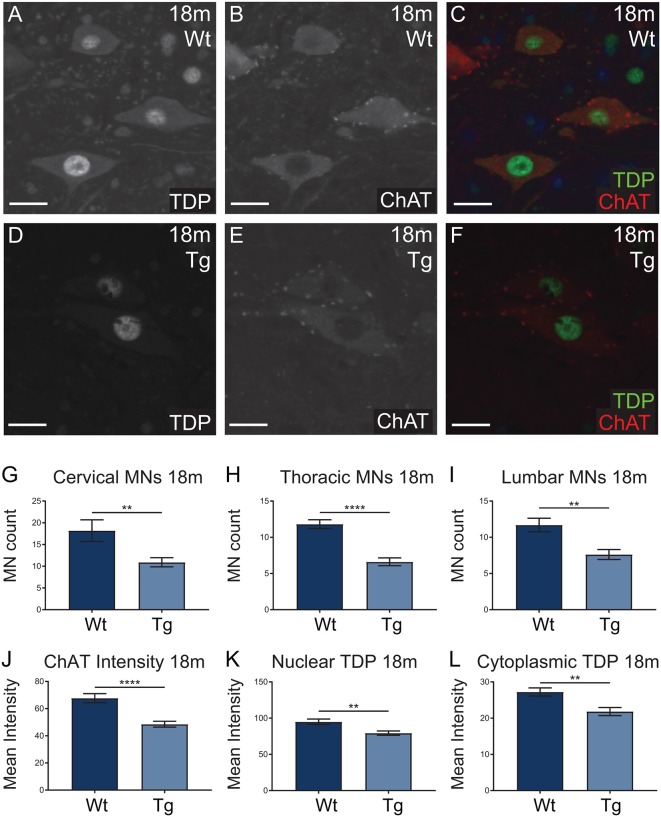
Wild type (Wt) controls and transgenic (Tg) sections from 18-month-old *Hb9* V5-PFN1^C71G^ mice were stained for ChAT (red) and TDP-43 (green; **A–F**). Scale bars = 20 μm. At 18-month-old, Tg mice had significantly fewer motor neurons per ventral horn of the spinal cord when compared to Wt controls **(G–I)**. This was consistent across the cervical, thoracic and lumbar regions of the spinal cord **(G–I)**. The motor neurons of Tg mice had significantly lower levels of ChAT intensity when compared with Wt controls **(J)**. At 18 months, Tg mice had significantly lower levels of nuclear and cytoplasmic TDP-43 in motor neurons **(K,L)**. Data were analyzed with unpaired student *t*-test; **p ≤ 0.01, ****p ≤ 0.0001; graphed with SEM. *N* = 3–5 mice, 11–41 sections or 81–97 cells.

*Hb9* V5-PFN1^C71G^ transgenic mice presented with significant motor deficits despite the lack of post-natal transgene expression. Next we analyzed the motor neurons in spinal cords to look for any molecular evidence of pathology. Spinal cords from 18-month-old mice were stained with ChAT (red; motor neuron marker) and TDP-43 (green), to assess motor neuron number and TDP-43 localization in motor neurons, respectively (Figures 8A–F). TDP-43 mis-localization is a key feature of ALS pathology (Tanaka et al., 2016; Fil et al., 2017). The number of ChAT positive motor neurons present in the cervical, thoracic and lumbar regions of spinal cords were analyzed in sections from 18-month-old mice. *Hb9* V5-PFN1^C71G^ transgenic mice had a significant reduction of motor neurons in all three regions when compared to Wt controls (Figures 8G–I). Analysis of the fluorescence intensity of ChAT in individual motor neurons revealed that 18-month-old old transgenic mice had a reduction in the levels of ChAT when compared to Wt controls (Figures 8J,K). The analysis of TDP-43 localization revealed that 18-month-old, normal weight male transgenic mice had a significant reduction in both cytoplasmic and nuclear levels of TDP-43 in motor neurons (Figures 8K,L). Aged *Hb9* V5-PFN1^C71G^ transgenic mice have some ALS-like features as they present with a reduced number of motor neurons and reduced ChAT staining intensity. They do not show evidence of a mis-localization of TDP-43 from the nucleus to the cytoplasm, instead, show a global reduction of TDP-43 in the nucleus and cytoplasm. Note that these changes in the spinal cord are present in 18-month-old transgenic mice, despite there being no transgene expression in adult mice.

## Discussion

The complex function of mutant PFN1 overexpression in neurons *in vitro* has previously been assessed, using cultured primary mouse hippocampal (Brettle et al., 2015) and motor neurons (Wu et al., 2012). Here, we specifically analyzed the impact of mutant PFN1 *in vivo* by α-motor neuron-specific expression of PFN1^C71G^. We have shown that the expression of ALS-associated PFN1^C71G^ in developing α-motor neurons has a lasting impact on the health and motor performance of transgenic mice. These effects persist long after the transgene expression has ceased. We confirmed that the expression of PFN1^C71G^ was consistent with previous studies using the *Hb9* promoter (Arber et al., 1999; Detmer et al., 2008; McGovern et al., 2015). This expression was mostly restricted spatially to developing α-motor neurons, and temporally to embryonic and neonatal transgenic mice. Expression of PFN1^C71G^ was observed from E10.5 and had decreased by P0. By 1 m of age, no transgene expression was detected in the brain or spinal cord of transgenic mice. Although Hb9 has been found in a small subset of gamma-motor neurons and spinal motor neurons in adult mice (Vult von Steyern et al., 1999; Chang and Martin, 2011), we saw no evidence of this in our mouse line. Transgene expression was not present in all ChAT positive motor neurons in transgenic embryos. This is consistent with other transgenic mouse lines that have pan-neuronal expression (van Hummel et al., 2018). This confirms that the phenotypes present in the *Hb9* V5-PFN1^C71G^ transgenic mice are a direct result of the developmental and neonatal expression of the ALS-associated PFN1 mutant.

PFN1 knock-out mice are embryonically lethal, this is likely due to the important role that PFN1 plays in cell division and migration (Witke et al., 2001; Kullmann et al., 2012, 2015). We showed that the expression of PFN1^C71G^ in developing motor neurons leads to the reduced diameter of brachial nerves in transgenic E14.5 embryos. This finding is consistent with *in vitro* reports that axonal outgrowth is reduced in cultured primary motor neurons, expressing PFN1^C71G^ (Wu et al., 2012). Axonal outgrowth is a dynamic process, for which PFN1 plays a key role. PFN1 localizes to the leading edge of growth cones, where it recruits actin monomers (Lee et al., 2013). Combined with the pivotal role that PFN1 plays in controlling the dynamics of actin and microtubules (Nejedla et al., 2016; Henty-Ridilla et al., 2017), we suggest that mutant PFN1^C71G^ has a loss of function in developing motor neurons. The synthetic PFN1^H120E^ mutant cannot bind to actin and reduces the recruitment of actin monomers to neuronal growth cones (Lee et al., 2013). If the PFN1^C71G^ also has a reduced capacity to recruit actin monomers to growth cones, this would be consistent with a loss of function hypothesis. The regulation of developmental nerves is an actin-dependent process. This is highlighted in a conditional knock-out of Rac1 in motor or sensory neurons. Rac1 knock-out in these neurons resulted in significantly thinner axonal bundles in fore and hind limbs (Hua et al., 2015). Rac1 and CDC42 are both Rho GTPases that are involved in actin dynamics and axonal outgrowth (Causeret et al., 2004; Schulz et al., 2016). An actin-deficient mutant of PFN1 suppressed CDC-42-dependent microspike formation and Rac1-dependent membrane ruffling (Suetsugu et al., 1998). We suggest that future studies focus on dissecting the impact that PFN1^C71G^ has at the growth cone with respect to the dynamics of actin and microtubules. Additionally, future studies should examine the impact of C71G mutation on the functional relationship between PFN1 and Rho GTPases.

Adult *Hb9* V5-PFN1^C71G^ transgenic mice presented with several distinct phenotypes. *Hb9* V5-PFN1^C71G^ mice have a Mendelian inheritance at embryonic stages, but not at the time of genotyping at 2 weeks old. We hypothesize that this is due to neonatal mortality, but further longitudinal studies would be needed to substantiate this. Weight and motor phenotypes were also identified. *Hb9* V5-PFN1^C71G^ transgenic mice weighed significantly less than the Wt controls at 1-month and 3-months. However, after 6 months of age, transgenic mice presented with a split weight phenotype consisting of a low weight and a normal weight group. The split weight phenotype could also be due to a metabolic change in the Hb9 V5-PFN1^C71G^ transgenic mice. Hb9 is expressed in the adult pancreas and knockout of Hb9 results in a reduction of the number of insulin-producing cells (Mu et al., 2016). This is particularly intriguing, given that diabetic gerbils have increased levels of PFN1 in liver tissue (Gong et al., 2018). Future studies characterizing the metabolic phenotype of the Hb9 V5-PFN1^C71G^ mice may provide further information about the role of PFN1 in the pancreas.

The 1-month and 3-month-old male transgenic mice and 3-month-old female transgenic mice presented with significant motor deficits when challenged on an accelerating Rotarod. From 6 months of age, normal weight transgenic mice presented with significant deficits when compared to Wt controls, but this was not consistent. In contrast to the normal weight transgenic group, the low weight transgenic mice that were older than 6 months did not present with significant motor deficits. The low weight transgenic mice were comparable to the Wt controls. A confounding factor is the reduction of the power of the statistical tests due to the split phenotype of the mice. Another factor was the reduction of mouse numbers over time, due to the reduced survival of *Hb9* V5-PFN1^C71G^ transgenic mice. Although young transgenic mice presented with motor deficits consistent with other ALS mouse models (Yang et al., 2016; van Hummel et al., 2018), the aged transgenic mice presented with a split motor phenotype which has not been previously reported. The split weight phenotype could be masking the motor phenotype in the low weight mice. Due to the relationship between motor performance and weight, lower weight mice would be predicted to perform better on Rotarod than heavier mice (Mao et al., 2015). The split weight phenotype could also indicate that the metabolic state of the transgenic mice is altered. A high body mass index has been implicated as a risk factor for ALS. Patient weight loss and hypermetabolism are associated with a decreased survival (Peter et al., 2017; Steyn et al., 2018). This, combined with the impact of animal weight on Rotarod performance, may contribute to the significant decrease in survival of low weight mice, despite the lack of an overt motor deficit. The expression of PFN1^C71G^ in developing motor neurons is sufficient to have a lasting impact on motor performance of *Hb9* V5-PFN1^C71G^ transgenic mice. However, this does not result in a full ALS phenotype.

The spinal cord analysis from aged 18-month-old transgenic mice revealed evidence of ALS-like pathology. There was a significant reduction in the number of ChAT-positive motor neurons throughout the spinal cords of 18-month-old transgenic mice. Furthermore, the levels of ChAT expression and global levels of TDP-43 expression were significantly reduced when compared to Wt controls. A reduction of ChAT expression is a hallmark of ALS and can be indicative of a loss of neuromuscular junctions (Casas et al., 2013; Fil et al., 2017). The reduction of ChAT expression in aged transgenic mice, combined with the reduced diameter of brachial nerve in transgenic embryos indicates that due to alterations in neuronal outgrowth, the circuitry of *Hb9* V5-PFN1^C71G^ transgenic mice may be irreparably altered. Although no cytoplasmic inclusions of TDP-43 were found in aged *Hb9* V5-PFN1^C71G^ transgenic mice, the reduction of TDP-43 expression levels in both the cytoplasm and nucleus of the motor neurons could impair the function of TDP-43. This reduction of TDP-43 could be due to several reasons, including increased clearance of TDP-43 or a downregulation of TDP-43 translation (Ke et al., 2015). The homozygous knock-out of TDP-43 is embryonically lethal and heterozygous knock-out mice with TDP-43 protein reduction have significant motor deficits (Kraemer et al., 2010). The downregulation of TDP-43 can induce neuronal death (Igaz et al., 2011). Taken together, the reduction of TDP-43 and ChAT levels is evidence of pathology in the aged *Hb9* V5-PFN1^C71G^ transgenic mice. This pathology is present despite no PFN1^C71G^ expression being present in adult *Hb9* V5-PFN1^C71G^ transgenic mice. Impaired neuronal health can lead to the reduction of neuromuscular junctions and eventually cell death. Aged transgenic mice presented with a significant reduction of motor neurons, supporting this possibility. Future studies should focus on the molecular cause of reduced TDP-43 in the aged *Hb9* V5-PFN1^C71G^ transgenic mice. This could be an important pathomechanism that links PFN1 mutations to other etiological causes of fALS.

The *Hb9* V5 PFN1^C71G^ transgenic mice differ in their phenotype from other *in vivo* models of ALS-associated PFN1 mutants (Yang et al., 2016; Fil et al., 2017). Although the motor deficits in young *Hb9* V5 PFN1^C71G^ transgenic mice emulated the motor deficits presented by Yang et al. (2016), our model did not progress to complete paralysis in aged mice. Furthermore, the model from Fil et al. (2017) showed cytoplasmic TDP-43 in α-motor neurons of transgenic mice, whereas aged *Hb9* V5 PFN1^C71G^ transgenic mice presented with an overall reduction of TDP-43. This finding further supports the link between PFN1 mutants and TDP-43 pathology, as our findings show that TDP-43 expression can be impacted in motor neurons long after transgene expression has ceased. This model of developmental expression of PFN1^C71G^ in motor neurons highlights the importance of PFN1 in development and in the pathogenesis of ALS.

The *Hb9* V5-PFN1^C71G^ transgenic mouse model shows a developmental phenotype that in part emulates existing *in vitro* and *in vivo* studies on PFN1^C71G^. Although additional studies are necessary to dissect the precise molecular mechanisms responsible for this developmental phenotype, the lasting impact of this phenotype impacts adult transgenic mice. Studies elaborating on the points of divergence in this complex profile of the *Hb9* V5-PFN1^C71G^ transgenic mouse model would aid in the understanding of ALS pathogenesis.

## Data Availability Statement

All datasets generated for this study are included in the manuscript/Supplementary Files.

## Ethics Statement

The animal study was reviewed and approved by University of New South Wales Animal Care and Ethics Committee.

## Author Contributions

TF, MB and LI designed the study. MB, HS, AD, SF, JC, AH, JH, MP and AV performed the experiments. MB analyzed all data. MB and TF wrote the manuscript. LI, YK and HS edited the manuscript. All coauthors read and confirmed the final manuscript. FD carried out the pronuclear injection for the generation of the mouse line. TF, YK and LI obtained funding. TF and LI supervised the project.

## Conflict of Interest

The authors declare that the research was conducted in the absence of any commercial or financial relationships that could be construed as a potential conflict of interest.
