# Supplementary material for: Developmental Expression of Mutant PFN1 in Motor Neurons Impacts Neuronal Growth and Motor Performance of Young and Adult Mice
Source: Front Mol Neurosci. 2019 Sep 27;12:231. doi: 10.3389/fnmol.2019.00231 (PMC6776973; doi:10.3389/fnmol.2019.00231)
Supplement: Supplementary file 1 [file Data_Sheet_1.PDF]

Supplementary Material

Supplemental Figure 1

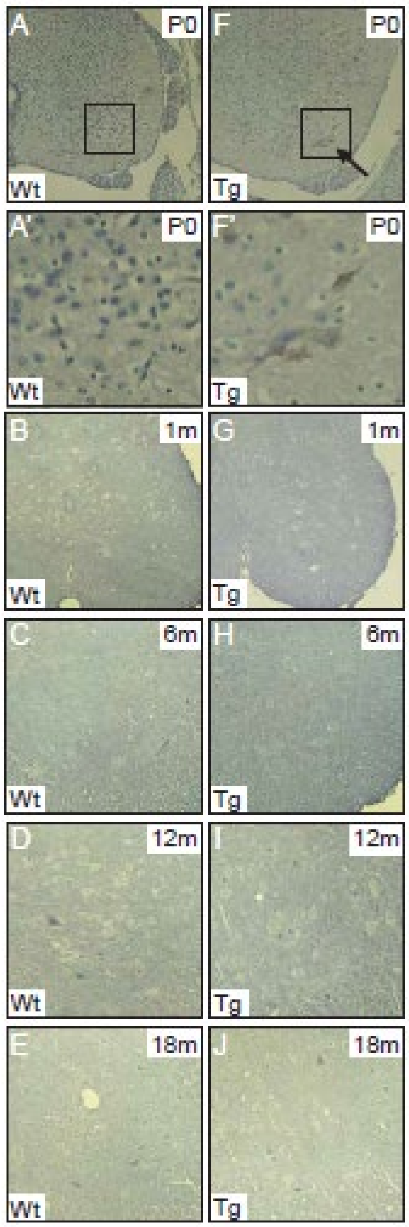

Supplementary Figure 1. Wild type controls (Wt) and transgenic (Tg) tissues were stained for V5-DAB. Spinal cords from Wt control have no expression of V5-PFN1^{C71G} (A-E). Cells in the anterior horn of the spinal cord in Tg P0 pups show limited V5-PFN1^{C71G} expression (F, Arrow, and zoomed area F'). There is no V5-PFN1^{C71G} expression in the spinal cords of adult Tg mice (G-J).
